# Microbiota-host interactions: key mechanisms linking periodontitis and systemic lupus erythematosus

**DOI:** 10.3389/fcimb.2026.1942138

**Published:** 2026-10-14

**Authors:** Xinyu Wu, Xinye Zhang, Yuang Guo, Ruiyi Zhang, Huiying Li, Wanghong Zhao

**Affiliations:** 1 Department of Periodontics, Stomatological Hospital, School of Stomatology, Southern Medical University, Guangzhou, China; 2 Department of Stomatology, Nanfang Hospital, Southern Medical University, Guangzhou, China; 3 Department of Stomatology, Xiamen Medical College, Xiamen, China

**Keywords:** autoimmune, dysbiosis, oral microbiota, periodontitis, systemic lupus erythematosus

## Abstract

Periodontitis is one of the most prevalent chronic inflammatory diseases in the oral cavity, characterized by progressive destruction of periodontal supporting tissues. Its pathogenesis involves complex interactions between the oral microbiota and host immune responses. This condition not only severely impacts oral health but is also closely associated with multiple systemic diseases. In recent years, the link between periodontitis and systemic lupus erythematosus (SLE) has garnered increasing attention. This review examines the compositional characteristics of the oral microbiota in patients with SLE and the epidemiological evidence linking periodontitis to SLE. It focuses on how periodontitis may trigger or exacerbate SLE’s autoimmune responses through the oral microbiota, via mechanisms such as molecular mimicry, cytokine-mediated immune activation, and microbial translocation, while elucidating the underlying pathophysiological and immunological mechanisms. Furthermore, this paper aims to explore potential prevention and treatment strategies based on oral microbial interventions, to provide novel insights and theoretical foundations for adjunctive therapy and disease management in SLE.

## 1 Introduction

Systemic lupus erythematosus (SLE) is an autoimmune disorder characterized by the production of autoantibodies and the formation of immune complexes. It exhibits significant clinical variability and can affect multiple organs, including the skin, joints, and kidneys (Siegel and Sammaritano, 2024). The complications associated with SLE can be severe, greatly impacting patients’ quality of life and long-term prognosis. The epidemiology of SLE varies across racial and ethnic groups and geographic regions, and the disease predominantly affects women, particularly those of reproductive age (Barber et al., 2021). Population-based registries in the United States reported the highest prevalence among American Indian/Alaska Native and Black women, with a substantial burden also observed among Hispanic and Asian/Pacific Islander women (Izmirly et al., 2021). The aetiology of SLE involves complex interactions among genetic, hormonal, and environmental factors (Tsokos, 2020). Although the precise mechanisms that initiate the loss of self-tolerance in SLE remain incompletely understood, impaired clearance and aberrant sensing of self-derived nucleic acids can activate plasmacytoid dendritic cells and other innate immune pathways, sustaining the production of type I interferons (IFN-I) and proinflammatory cytokines; concurrently, dysregulated T- and B-cell responses promote autoantibody production. The resulting autoantibodies form immune complexes with self-antigens; deposition of these complexes in tissues activates complement- and Fc receptor-mediated pathways, driving inflammatory organ damage (Tsokos, 2020).

Periodontitis is a chronic inflammatory disease caused by plaque microorganisms, characterized by progressive destruction of periodontal supporting tissues due to dysregulated host immune responses (Williams et al., 2021). It constitutes the primary cause of adult tooth loss, not only inducing oral dysfunction but also being recognized as a significant risk factor for multiple systemic diseases (Isola et al., 2025). The onset and progression of periodontitis depend on the dynamic interaction between microbial infection and host immune-inflammatory responses.

The oral cavity is the second largest microbial habitat in the human body, and the oral microbiome plays a crucial role in maintaining both oral and systemic health (Baker et al., 2024). Under healthy conditions, oral microbes coexist in a state of dynamic equilibrium with the host. However, under disease conditions, the oral microbial community may become unbalanced, a condition termed dysbiosis, characterized by the expansion of disease-associated microorganisms and depletion of health-associated commensals. For example, periodontitis-associated bacteria such as *Porphyromonas gingivalis*(*P. gingivalis*), *Tannerella forsythia* and *Treponema denticola* may increase, whereas health-associated commensals and early colonizers such as *Streptococcus sanguinis* and *Actinomyces* spp. may decrease (Curtis et al., 2020). This shift exacerbates local inflammation and may have effects on distant organs (Kobayashi and Bartold, 2023).

Recent evidence indicates that the prevalence and severity of periodontitis in patients with SLE are significantly greater than those in healthy individuals, suggesting a potential pathophysiological link between the two conditions (Rutter-Locher et al., 2017; Wu et al., 2017; Gofur et al., 2019; Bolstad et al., 2022; Tan et al., 2024). Genetic susceptibility to SLE is largely polygenic and involves the major histocompatibility complex, as well as pathways regulating nucleic acid sensing, IFN-I signalling, B-cell tolerance, nuclear factor-κB (NF-κB) activity, and immune-complex clearance; rare deficiencies in early components of the classical complement pathway confer strong monogenic susceptibility (Ghodke-Puranik et al., 2024). *FCGR2A/FCGR2B* genotypes and the *DNMT3B* rs2424913 polymorphism have been associated with periodontitis in patients with SLE, supporting a shared host-genetic contribution to the clinical overlap between the two diseases (Kobayashi et al., 2003, 2007; Dias et al., 2022). Beyond host susceptibility, the oral cavity is a key interface between the human body and the environment, and the balance of its microbiota is essential for maintaining local and systemic immune homeostasis. When this balance is disrupted, the overgrowth of specific pathogenic bacteria can trigger local oral inflammation and may also initiate or worsen systemic autoimmune responses. This review aims to systematically characterize the oral microbiota in SLE patients, summarize the epidemiological and clinical evidence linking SLE with periodontitis, and explore potential biological mechanisms involved. These mechanisms may include pathways such as cytokine amplification of autoimmunity, molecular mimicry triggering autoimmunity, and microbial translocation. Additionally, this review will address the importance of intervention strategies that target the oral microbiome as part of a comprehensive management plan for SLE and outline future research directions in this area.

## 2 The link between SLE and periodontitis

Periodontitis, a common disease affecting the supporting structures of the periodontal tissue, is closely associated with microbial dysbiosis in its development. Dynamic imbalances in the oral microbiota may trigger immune-inflammatory cascades, which are key factors in the destruction of periodontal tissues (Curtis et al., 2020).

Multiple systematic reviews and meta-analyses have provided substantial evidence for the association between SLE and periodontitis. Compared with healthy controls, patients with SLE exhibit a significantly increased risk of developing periodontitis, with an odds ratio (OR) of 1.76 (95% CI: 1.29–2.41) (Rutter-Locher et al., 2017). Similarly, in the study by Zhong et al (Zhong et al., 2020), with healthy controls as the reference, the OR was 5.32 (95% CI: 1.69–16.78). A recent meta-analysis compared the risk of periodontitis between patients with SLE and those with rheumatoid arthritis (RA) (Tan et al., 2024). RA is recognized as a rheumatic autoimmune disorder closely linked to periodontitis (Kobayashi and Bartold, 2023). This analysis found a higher prevalence of periodontitis in SLE patients compared to RA patients (OR = 1.49, 95% CI: 1.09–2.05). However, in a subgroup analysis focused on female patients, the association between SLE and periodontitis did not reach statistical significance (OR = 3.20, 95% CI: 0.85–12.02) (Hussain et al., 2022). This finding suggests that gender may influence the strength of this association, indicating a need for further investigation into potential underlying mechanisms. Mendelian randomization studies provide additional genetic evidence supporting the causal relationship between SLE and periodontitis. Bae and Lee, 2020 found that periodontitis may contribute to SLE pathogenesis, suggesting that oral microbiota dysbiosis may be involved in SLE development rather than merely being a consequence of it. Another bidirectional Mendelian randomization (MR) study demonstrated that SLE may increase the risk of periodontal disease (defined as including both periodontitis and gingivitis in that study) (Inverse Variance Weighted [IVW] method: OR = 1.079, P < 0.001), while periodontal disease may also decrease the risk of SLE (IVW: OR = 0.808, 95% CI: 0.676–0.965) (Zhang et al., 2024a). Both interventional and cross-sectional observational studies support these conclusions (**Table 1**). For example, Fabbri et al., 2014 found that periodontal treatment improved the response to immunosuppressive therapy in SLE patients, significantly reducing disease activity following periodontal intervention. Multiple observational studies have highlighted that the prevalence of periodontitis is significantly higher in patients with SLE compared with age- and sex-matched healthy controls, and that periodontitis is associated with exacerbation of SLE (Wu et al., 2017; Zhang et al., 2017; Zamora-Pasadas et al., 2022).

**Table 1:** Characteristics of clinical studies investigating the association between periodontitis and SLE.

Subjects	Study design	Region	Mean age (years)	% female	% smokers	Other influencing factors	Main findings	Reference
SLE-T=32 SLE-NT=17	Randomized controlled trial	Brazil	SLE-T =32.7 SLE-NT=29.1	SLE-T =78.1 SLE-NT=94.1	NR	NR	Periodontal treatment improved the response to immunosuppressive therapy and significantly reduced disease activity in SLE patients.	(Fabbri et al., 2014)
SLE=25 CON=50	Case-control	Kingdom of Saudi Arabia	SLE=33.32 CON=36.73	SLE=100 CON=100	SLE=0 CON=0	Vitamin D levels, rheumatologic parameters, SLE-related clinical manifestations	No significant difference in periodontal health was found between SLE patients and controls; however, in SLE patients, flare-ups and arthritis were significantly linked to periodontal status.	(Al-Mutairi et al., 2015)
SLE=75 CON=75	Case-control	Brazil	SLE=38 CON=41	SLE=91 CON=77	SLE=11 CON=11	Diabetes mellitus, educational level, income, race	No significant difference in periodontitis frequency between SLE and controls, but SLE patients developed periodontitis at a younger age.	(Calderaro et al., 2017)
SLE=108 CON=108	Case-control	China	SLE=37.48 CON=39.05	SLE=100 CON=100	NR	Educational level, urban/rural residence, BMI, SLE duration, SLEDAI, rheumatologic parameters, medication use	SLE patients had higher risks of periodontitis and edentulism than controls. Periodontitis was associated with age, lower education, higher BMI, disease activity, and prednisone use, but age was the only independent predictor.	(Zhang et al., 2017)
SLE=7204 CON=72040	Case-control	China	SLE=40 CON=40	SLE=86 CON=86	NR	Diabetes mellitus, periodontitis treatment history	Periodontitis history was significantly associated with incident SLE, and the association was consistent across age, gender, and diabetes subgroups.	(Wu et al., 2017)
SLE=75 CON=78	Case-control	Brazil	SLE=38.03 CON=41.31	SLE=90.7 CON=79.5	SLE=21.3 CON=25.6	Educational level, household income, oral hygiene habits, salivary flow rate	SLE patients showed no significant difference in periodontitis prevalence compared with controls, but had more missing teeth.	(Corrêa et al., 2018)
SLE=61	Cross-sectional	Indonesia	SLE=29.47	SLE=100	SLE=0	SLE-related clinical manifestations, disease duration, SLEDAI, rheumatologic parameters	Periodontitis scores show a strong positive association with SLEDAI, anti-dsDNA, and IFN-γ, suggesting that they could serve as a dual biomarker for SLE disease activity and immune aging.	(Gofur et al., 2019)
SLE=1990 CON=170332	Case-control	Norway	NR	SLE=90.8 CON=51.1	NR	Myocardial infarction, diabetes mellitus	SLE patients showed nearly a twofold risk of periodontitis relative to controls, with the disparity being most pronounced among younger patients.	(Bolstad et al., 2022)
SLE=20 CON=20	Case-control	Iran	SLE=38.65 CON=38.30	SLE=60 CON=55	SLE=0 CON=0	Salivary flow rate and pH	SLE patients had similar periodontal status to controls, but more missing teeth, possibly due to caries.	(Bashiri et al., 2026)
SLE=114 ORD=228	Cross-sectional	Peru	Total=48.89	SLE=100 ORD=100	SLE=22 ORD=10	Medication use, hypertension, diabetes mellitus	No overall association was found between periodontitis and SLE; however, correlations were observed in specific subgroups (aged 27–59 years, non-smokers, and those without hypertension or diabetes).	(Bocanegra-Baez et al., 2026)

SLE, systemic lupus erythematosus; SLE-T, SLE patients who receive periodontal treatment; SLE-NT, SLE patients who did not receive periodontal treatment; CON, controls; ORD, other rheumatic diseases; SLEDAI, Systemic Lupus Erythematosus Disease Activity Index; NR, not reported; BMI, body mass index; anti-dsDNA, anti-double-stranded DNA antibody; IFN-γ, interferon-gamma.

However, individual studies have not consistently found a significant association between SLE and periodontal disease. For instance, Al-Mutairi et al., 2015 and Calderaro et al., 2017 reported no significant differences in periodontal health between patients with SLE and healthy controls. These studies have been cross-sectional surveys with limited sample sizes, which provide weaker evidence compared to large-scale meta-analyses or MR studies. Interestingly, one early study even suggested that periodontal probing depths were significantly lower in SLE patients compared to healthy individuals (Mutlu et al., 1993). This inconsistency may be influenced by the effects of pharmacological treatments. Many patients with SLE are on long-term medication, including glucocorticoids, immunosuppressants, or biologics. These treatments have strong anti-inflammatory and immunomodulatory effects, which may mask clinical signs of periodontal inflammation and delay alveolar bone loss. As a result, clinical indicators of periodontal health in SLE patients might resemble those of healthy individuals, despite the possibility of underlying subgingival microbial imbalances. Furthermore, existing studies show considerable heterogeneity in key demographic and clinical characteristics, including age and sex distribution, geographic region, smoking status, medication exposure, and the extent of adjustment for potential confounders. Such variability is likely a major contributor to the inconsistent findings reported across studies.

These studies provide initial epidemiological evidence suggesting a potential association between SLE and periodontitis. However, most conclusions remain limited to the aggregation of cross-sectional data and do not explore the underlying mechanisms or causal directions that drive the observed associations. Future research should focus on a more critical scientific question: having established the correlation, how can we progress from asking ‘whether an association exists’ to understanding ‘how it is established’? We advocate for future studies to move beyond simply validating replicability and instead conduct higher-level evidence through interventional randomized controlled trials and prospective cohort studies. For example, researchers could recruit cohorts comprising individuals with preclinical SLE, newly diagnosed SLE patients, genetically susceptible high-risk individuals, unaffected first-degree relatives, or cohabiting family members (whose oral microbial compositions may be similar), along with healthy controls, and conduct long-term follow-up. Through regular standardized periodontal clinical examinations and oral microbiome sampling, the temporal relationship and predictive value of oral dysbiosis in relation to SLE onset can be systematically assessed across diverse genetic backgrounds and environmental exposures. Furthermore, conducting periodontal intervention therapy in SLE patients, using oral hygiene instruction alone as a control, can provide valuable insights. Changes in the Systemic Lupus Erythematosus Disease Activity Index (SLEDAI), serum inflammatory cytokines, autoantibody titers, and the oral microbiome should be assessed at baseline, immediately after the intervention, and at multiple follow-up time points. This approach will yield higher-level causal evidence supporting the proposition that ‘treatment of periodontitis improves SLE prognosis’ and will help elucidate the dynamic mechanisms underlying its effectiveness.

## 3 Oral microbiota characteristics in SLE patients

In recent years, an increasing number of studies have focused on the oral microbiota characteristics of SLE patients (**Table 2**). In 1999, Jensen et al., 1999 first compared the oral microbial composition of SLE patients and healthy controls, thereby initiating investigations into oral microbiome in this population.

**Table 2:** Characteristics of clinical studies investigating oral microbiota alterations in SLE patients.

Subjects	Sample	Method	Mean age (years)	% female	% medication	% smokers	Antibiotics (exclusion)	Other influencing factors	Diversity analysis	Main findings	Reference
SLE=40 CON=40	Stimulated saliva	Plate Count	SLE=33.1 CON=32.8	SLE=100 CON=100	SLE=100 CON=0	SLE=0 CON=0	Used within 2 months	Salivary secretion rate	NR	No significant difference in oral microbial counts was observed between SLE patients and healthy controls.	(de Araújo Navas et al., 2012)
ISLE=30 ASLE=30	Stimulated saliva	qPCR	ISLE=31.0 ASLE=32.0	ISLE=93 ASLE=93	NR	NR	Used within 3 months	Salivary variables: flow rate, pH, and buffering capacity; SLE duration; Monthly income	NR	Dental caries frequency was higher in ASLE than in ISLE and was associated with salivary flow rate, pH and buffering capacity and with cariogenic bacterial counts.	(Loyola Rodriguez et al., 2016)
CON+Non-CP=24 CON+CP=28 SLE+Non-CP=17 SLE+CP=35	Subgingival plaque	16S rRNA V4	CON+Non-CP=34.7 CON+CP=46.3 SLE+Non-CP=31.2 SLE+CP=40.5	CON+Non-CP=29 CON+CP=48 SLE+Non-CP=30 SLE+CP=57	NR	CON+Non-CP=3.81 CON+CP=7.60 SLE+Non-CP=5.70 SLE+CP=9.60	Used within 3 months	Oral hygiene habits; SLE duration	α: Chao1, Observed sp.; β: UniFrac (weighted & unweighted)	SLE patients had earlier and more prevalent periodontitis, with enrichment of specific pathogens, and elevated local inflammatory cytokines.	(Corrêa et al., 2017)
SLE=20 CON=20	Unstimulated saliva	DGGE; qPCR	SLE=42.4 CON=43.0	SLE=90 CON=90	SLE=0 CON=0	NR	Used within 1 week	Oral hygiene habits; Periodontal status; Salivary variables: flow rate, pH, and buffering capacity	α: Shannon	Salivary dysfunction accompanied an altered oral microbiota: *S. mutans* and S*. sobrinus* increased while *S. sanguinis* and *S. gordonii* decreased.	(Yang et al., 2018)
SLE=34 pSS=34	Buccal swab; Oral wash	16S rRNA V4	SLE=48.0 pSS=54.0	SLE=88.2 pSS=94.1	NR	SLE=26.5 pSS=8.8	Used within 2 months	Ethnicity, Country of birth, Disease duration, Xerostomia score, Self-reported oral health	α: Shannon, Chao1; β: Bray-Curtis	Despite a largely shared gut microbiota, SLE and pSS patients had distinct oral microbiota.	(Van Der Meulen et al., 2019)
SLE=20 CON=19	Buccal swab	16S rRNA V4-V5	SLE=31.6 CON=29.6	NR	NR	NR	NR	Diet	α: Chao1, ACE, PD whole tree, Shannon, Simpson, sob; β: UniFrac (weighted & unweighted), Jaccard	SLE patients exhibited reduced oral microbial diversity, enrichment of Lactobacillaceae and Veillonellaceae, depletion of Sphingomonadaceae and other families, and aberrant metabolic pathways.	(Li et al., 2020)
SLE=35 CON=35	Unstimulated saliva	16S rRNA V3-V4	NR	NR	SLE=85.7 CON=0	NR	Used within 2 weeks	SLE duration	α: Chao1, ACE, Shannon, Simpson; β: Bray-Curtis	SLE patients showed higher salivary microbial diversity than healthy controls, dominated by *Veillonella*, *Streptococcus*, and *Prevotella*, with *Bacteroides* negatively correlated with SLEDAI.	(Liu et al., 2021)
SLE=100 CON=200	Tongue coating	16S rRNA V3-V4	SLE=34.0 CON=32.0	SLE=94 CON=92	SLE=0 CON=0	NR	Used within 1 month	NR	α: Shannon, Simpson; β: NMDS, PCoA, PCA	SLE patients exhibited increased microbial diversity in tongue coating (*Prevotella/Veillonella*↑, *Streptococcus/Porphyromonas*↓). The oral microbiota may serve as a non-invasive adjunctive diagnostic biomarker for SLE patients following treatment.	(Guo et al., 2023)
SLE=30 RA=31 CON=30	Supragingival plaque	Plate count	NR	NR	NR	NR	Used within 1 month	Education level, Marital status, Dental caries status	NR	SLE and RA patients had higher counts of *Streptococcus mutans*, *Lactobacillus*, and *Candida albicans* in supragingival plaque and more severe dental caries than healthy controls; SLEDAI-2K positively correlated with *Candida albicans* and *Lactobacillus* counts.	(Mehdipour et al., 2025)

SLE, systemic lupus erythematosus; CON, controls; ISLE, inactive systemic lupus erythematosus; ASLE, active systemic lupus erythematosus; pSS, primary Sjögren’s syndrome; RA, rheumatoid arthritis; SLEDAI, SLE disease activity index; Non-CP, non-periodontitis; CP, periodontitis; NR, not reported; DGGE, denaturing gradient gel electrophoresis; qPCR, quantitative polymerase chain reaction; SLEDAI-2K, Systemic Lupus Erythematosus Disease Activity Index 2000; NMDS, non-metric multidimensional scaling; PCoA, principal coordinate analysis; PCA, principal component analysis; ACE, abundance-based coverage estimator; PD, phylogenetic diversity.

### 3.1 Changes in microbial diversity

Regarding alterations in oral microbial diversity among SLE patients, existing research findings are not entirely consistent. Liu et al., 2021 observed increased α-diversity of salivary microbiota and decreased α-diversity of faecal microbiota in Chinese SLE patients compared to healthy controls. This aligns with Guo et al.’s findings, who measured Shannon and Simpson indices to demonstrate significantly elevated oral microbial α-diversity in untreated SLE patients, which persisted even after stabilization following treatment (Guo et al., 2023). In contrast, Li et al., 2020 and Yang et al., 2018 reported reduced oral microbial diversity in SLE patients. Corrêa et al., 2017 reported that subgingival microbiome α-diversity was significantly higher in SLE patients than in healthy controls at healthy gingival sites, but lower in SLE patients than in healthy controls at sites with periodontal inflammation.

Several factors may explain this contradiction: Firstly, differences in sample types and sites: Significant variations in sample sources (e.g., saliva, gingival crevicular fluid, tongue coating, or mucosal swabs) across studies may arise, as microbial community structures exhibit site-specificity (Mark Welch et al., 2020). Direct comparisons between such samples may introduce bias. Secondly, there is the issue of patient cohort heterogeneity. Key variables such as disease activity, medication history (including antibiotic, corticosteroid, or immunosuppressant use), periodontal status, and the presence of other autoimmune diseases may not have been appropriately matched or adjusted for in different studies. For instance, Li et al., 2020 included both untreated and treated patients, where therapeutic agents could confound microbial composition. Corrêa et al., 2017 enrolled SLE patients with secondary Sjögren’s syndrome (sSS), while the effects of primary Sjögren’s syndrome on oral microbiota are well-known; the specific impact of sSS remains unclear (Van Der Meulen et al., 2019). Thirdly, methodological variations also play a role. The choice of variable regions in 16S rRNA gene sequencing, sequencing depth, and bioinformatics analysis workflows can influence diversity assessments. For example, Yang et al., 2018 used Denaturing Gradient Gel Electrophoresis (DGGE) technology to estimate species richness, a method characterized by low resolution, an inability to detect rare species, and poor quantitative accuracy. This approach may underestimate the true community diversity and struggle to capture structural differences comprehensively. Finally, inadequate control of confounding factors can skew results. Factors such as smoking (Dai et al., 2025), dietary patterns (Zhang et al., 2025), ethnicity (Wang et al., 2024), and salivary physiological indicators (Nagakubo and Kaibori, 2023) can independently affect oral microbiota composition. However, most studies fail to incorporate these variables into multivariate models or stratified analyses, which may contribute to the inconsistent results observed across studies.

In summary, future research on microbial diversity needs to standardize sampling sites and methods rigorously. It is essential to document patients’ medication histories, dietary habits, and oral health conditions meticulously. Employing more precise and comprehensive sequencing analyses whenever possible is also crucial. Only by adhering to such standardized protocols can we effectively elucidate the intrinsic effects of SLE on the oral microbiome.

### 3.2 Changes in abundance of specific genera within oral microbiota

#### 3.2.1 Genera with increased abundance

Numerous studies have indicated a significant increase in the abundance of several bacterial genera within the oral microbiome of patients with SLE. These changes may be closely linked to the immune inflammatory state of SLE and its impact on periodontal health.

*Prevotella* species are commonly found in increased levels in the saliva and subgingival plaque of SLE patients. Notable species include *Prevotella oulorum*, *Prevotella pleuritidis*, and *Prevotella oris* (Corrêa et al., 2017; Liu et al., 2021; Guo et al., 2023). Importantly, the abundance of *Prevotella denticola* (*P. denticola*) in subgingival plaque has shown a significant positive correlation with serum IL-33 levels in these patients (Corrêa et al., 2017). IL-33 is a multifunctional alarmin released upon tissue injury (Li et al., 2026). Its levels are elevated in patients with SLE and correlate positively with disease activity (Lu et al., 2025). Mechanistically, IL-33 induces BAFF expression, which promotes B-cell differentiation and autoantibody class switching, thereby breaking immune tolerance and amplifying autoimmunity (Rose et al., 2018). The association between a subgingival pathobiont and this systemic alarmin is therefore biologically plausible. It nonetheless derives from a single cross-sectional study, and the design precludes any inference as to whether microbial enrichment drives the cytokine response, follows it, or is simply a parallel marker of periodontal inflammation.

*Veillonella* predominates the salivary microbiome in SLE patients, particularly showing marked elevation in newly diagnosed cases or those with oral ulcers, indicating significant dysbiosis (Li et al., 2020; Liu et al., 2021; Guo et al., 2023). As a key commensal involved in early oral colonization and biofilm formation, this imbalance is associated not only with dental caries and periodontal disease but may also facilitate transmucosal migration for ectopic colonization, thus promoting systemic inflammatory responses (Schmidt et al., 2019).

The relative abundance of *Selenomonas* has significantly increased as well (Corrêa et al., 2017; Liu et al., 2021). Its levels in subgingival plaque are positively correlated with serum IL-6 and IL-33 (Corrêa et al., 2017). As a periodontal pathogen, elevated levels of *Selenomonas noxia* may indicate a transition from a healthy microbiome to an inflammatory state, suggesting potential subclinical or early periodontal lesions (McDaniel et al., 2021). This bacterium remains significantly enriched in periodontally healthy SLE patients, reinforcing the idea that disruption in the oral microbiome can occur prior to the typical clinical manifestations of periodontal disease in SLE patients (Corrêa et al., 2017).

*Lactobacillus* is also significantly enriched in the oral cavity of SLE patients, with its levels correlating with disease severity (Li et al., 2020; Liu et al., 2021). Higher levels of this genus are observed in the saliva of patients with moderate to severe active disease (Liu et al., 2021). Furthermore, studies have identified a negative correlation between the abundance of oral *Lactobacillus* and intestinal *Actinomyces* in SLE patients. This suggests that the oral microbiota may act as a reservoir for potential pathogens associated with intestinal inflammation, which could in turn influence distal microbial composition and immune homeostasis via the oral-gut axis (Van Der Meulen et al., 2019).

In addition to the aforementioned bacterial genera, *Treponema*, *Barnesiella*, *Blautia*, *Pyramidobacter*, and *Leptotrichia* also exhibited consistent enrichment in the oral cavities of SLE patients, further corroborating the extensive and complex nature of oral microbiota dysbiosis in SLE (Corrêa et al., 2017; Li et al., 2020; Liu et al., 2021; Guo et al., 2023).

#### 3.2.2 Genera with decreased abundance

In the oral microbiome of SLE patients, a notable decline in specific common commensal genera has been observed, indicating a disruption of the microbial ecosystem.

*Streptococcus* species were generally reduced by 6–8% in SLE patients (Corrêa et al., 2017; Liu et al., 2021; Guo et al., 2023). In particular, several *Streptococcus* species, including *Streptococcus sanguinis* and *Streptococcus gordonii*, showed significant decreases in abundance. These strains are early colonizers of the oral cavity and are essential for maintaining microbial balance and inhibiting the growth of pathogenic bacteria (Yang et al., 2018; Liu et al., 2021). Conversely, the proportions of *Streptococcus mutans* (*S. mutans*) and *Streptococcus sobrinus*, which are associated with dental caries, were notably elevated (Yang et al., 2018). This shift suggests an ecological imbalance in the oral cavity of SLE patients, with an increase in acid-producing cariogenic bacteria and a decrease in beneficial commensals, possibly contributing to the higher prevalence of dental caries observed clinically (Gofur et al., 2020).

Additionally, genera typically considered part of the normal oral microbiota, such as *Rothia*, *Haemophilus*, *Actinomyces*, and *Granulicatella*, have been reported to have reduced abundance in SLE patients in some studies (Corrêa et al., 2017; Li et al., 2020; Liu et al., 2021; Guo et al., 2023). This further indicates a widespread lack of protective symbiotic microorganisms in the oral microbiota of SLE patients, which may impair ecological function.

#### 3.2.3 Synthesis and perspectives

In summary, existing research has clearly shown that the oral microbiome of SLE patients exhibits ecological dysregulation, characterized by an increase in pathogenic bacteria and a decrease in commensal species. These findings provide an important foundation for understanding the oral microenvironment in SLE. However, changes in microbial diversity and relative abundance alone cannot fully account for the biological consequences of oral dysbiosis. Certain microorganisms, even at low abundance, may exert large effects on community structure and host immune homeostasis (Hajishengallis et al., 2012). For example, *P. gingivalis* has been described as a key periodontal pathogen capable of disrupting host immune surveillance and reshaping the originally commensal microbial community into an inflammatory, dysbiotic community (Hajishengallis and Lamont, 2012). Thus, beyond conventional abundance-based analyses, greater attention should be directed toward functional interactions among microorganisms and their immunomodulatory effects on the host. For example, how do enriched genera such as *Prevotella* and *Veillonella* interact with depleted genera like *Streptococcus* and *Rothia*? How does this specific community structure ultimately affect immune pathways or correlate with different clinical subtypes of SLE? To address these questions, future research needs to integrate multi-omics technologies, such as metagenomics and metabolomics, along with animal models. This approach will help move from simply observing correlations to establishing causation. Ultimately, this will allow for the creation of pathogenic network models involving the oral microbiota in SLE, providing a theoretical basis for targeted microbial interventions.

### 3.3 The potential of the oral microbiota as a biomarker for SLE

The potential of the oral microbiome as a non-invasive biomarker has been validated across multiple diseases (Zhang et al., 2015; Qiu et al., 2024; Li et al., 2025). Guo et al., 2023 demonstrated a significant association between oral microbiota and multiple clinical indicators of SLE, including gender, age, white blood cell count, hemoglobin, platelet count, lymphocyte count, and globulin. They developed a diagnostic model for SLE that utilizes oral microbiota, which showed robust efficacy when validated in an independent cohort. Significant correlations have been observed between the abundances of specific bacterial genera and disease activity. For instance, *Bacteroides* abundance negatively correlates with the SLEDAI, while *Staphylococcus* abundance positively correlates with SLEDAI scores (Liu et al., 2021). Li et al., 2020 identified a panel of oral microbiota-derived biomarkers, including the phyla *Actinobacteria* and *Tenericutes*, along with the genera *Barnesiella*, *Blautia*, *Lactobacillus*, *Pyramidobacter*, and *Veillonella*. The diagnostic model achieved an area under the curve (AUC) of 0.953, indicating excellent discriminatory power in distinguishing SLE patients from healthy individuals, with an estimated 95.3% probability of correct classification. However, their sensitivity and specificity have not been validated against established gold standards. Additional serological evidence supports the diagnostic value of oral microbiota. For example, using an enzyme-linked immunosorbent assay, Bagavant et al., 2019 observed a significant correlation between elevated levels of anti-*Aggregatibacter actinomycetemcomitans (A. actinomycetemcomitans)* antibodies and increased SLEDAI scores. A recent case-control study further found that anti-*A. actinomycetemcomitans* LtxA antibody levels were significantly associated with anti-dsDNA antibody positivity, further supporting a specific link between oral microbial exposure and SLE-related autoimmunity (Massarenti et al., 2025). These studies suggest that future research could utilize multi-omics technologies to develop stratified diagnostic and therapeutic strategies for SLE based on oral microbiota biomarkers.

Beyond diagnostic classification, an important future direction is the development of prognostic and predictive oral microbiome biomarkers. Longitudinal studies should determine whether oral microbial signatures can predict SLE development in at-risk individuals, subsequent disease flares or progression, target-organ involvement, or response to immunomodulatory therapy. Such applications may offer greater clinical value than merely distinguishing established SLE from healthy states.

## 4 Potential microbiota-mediated mechanisms linking periodontitis and SLE

### 4.1 Cytokine-driven amplification of autoimmunity

In recent years, multiple studies have suggested that oral dysbiosis and periodontitis may contribute to the immunopathogenesis and progression of autoimmune diseases by modulating cytokine networks. Both SLE and periodontitis are chronic inflammatory diseases characterised by abnormal B-cell activation, dysregulated pro-inflammatory cytokine secretion, and persistent immune response activation (Enocsson et al., 2020; Iwasaki et al., 2025). During this process, various cytokines may mutually induce and act synergistically, thereby potentially further exacerbating local periodontal tissue destruction and promoting a systemic autoimmune response. However, most conclusions in the field of SLE and oral microbiota remain grounded in clinical studies, with causal relationships and clinical translational value requiring further validation.

At the molecular level, periodontal pathogens and their metabolic products are thought to activate innate immune cells through pattern recognition receptors, such as Toll-like receptors (Engevik et al., 2021; Oner et al., 2025). This activation leads to the release of key inflammatory mediators, including interleukin (IL)-1β, IL-6, tumor necrosis factor-α (TNF-α), and IL-17 (Schulze-Späte et al., 2024). Among these, IL-1 enhances immune cell activation and intensifies the effects of TNF-α and IL-6, resulting in increased tissue damage in patients with SLE (Elemam et al., 2025). Meanwhile, IL-6 promotes the differentiation of B cells into plasma cells, accelerating the production of autoantibodies (Tackey et al., 2004). In the context of inflammatory and autoimmune diseases, IL-17 works synergistically with TNF-α, IL-1β, and interferon-γ (IFN-γ) to enhance the NF-κB and mitogen-activated protein kinase (MAPK) signaling pathways, which in turn promotes the expression of additional inflammatory mediators (Onishi and Gaffen, 2010). Notably, research conducted by Corrêa et al., 2017 revealed that the presence of certain bacterial genera, including *Selenomonas*, *Prevotella denticola*, *Veillonella atypica*, and *Leptotrichia*, in the subgingival plaque of SLE patients was positively correlated with elevated salivary levels of IL-6, IL-17, and IL-33. This suggests that specific oral microbiota may act as immunomodulatory triggers involved in SLE disease activity. It’s important to emphasize that this evidence comes from cross-sectional correlational studies, which have not demonstrated temporal or causal relationships between microbial enrichment and changes in cytokine levels. Additionally, the findings may be influenced by confounding factors, such as drug therapy and the heterogeneity of the samples studied.

Beyond traditional inflammatory mediators, novel immunomodulatory substances such as visfatin have gained attention. As a pro-inflammatory adipokine, visfatin stimulates monocytes to produce IL-1β, TNF-α, and IL-6, while also regulating B-cell proliferation and antibody production (Dakroub et al., 2021; Semerena et al., 2023). Clinical studies have shown significantly elevated levels of visfatin in the gingival crevicular fluid of SLE patients with periodontitis, and these levels are positively correlated with SLEDAI scores (Rezaei et al., 2019). This suggests that visfatin may play a bridging role linking local oral inflammation to systemic SLE disease activity. However, relevant human studies often suffer from limited sample sizes, and the mechanisms are primarily derived from *in vitro* experiments that lack *in vivo* functional validation and support for interventional research. Therefore, the clinical significance of visfatin requires careful evaluation.

B-cell-activating factor (BAFF), a pivotal regulator of B-cell survival, maturation, and autoantibody production, plays a central role in the pathogenesis of SLE (Vincent et al., 2014) (Giordano et al., 2023). Clinical studies reveal that serum BAFF levels in SLE patients correlate significantly not only with periodontal clinical indicators (such as probing depth and clinical attachment loss) but also with disease duration and complement C3 levels (Zhang et al., 2022). This suggests that periodontal inflammation may promote the activation of auto-reactive B cells and the production of pathogenic autoantibodies by upregulating BAFF expression. This finding, however, remains correlative and does not elucidate the specific molecular pathways through which periodontal microbiota regulate BAFF expression, representing an important direction for future mechanistic research.

The findings discussed above underscore the importance of cytokine-mediated immune amplification mechanisms in the link between periodontitis and SLE. However, most current evidence is based on correlational studies, making it difficult to establish causal relationships and identify specific molecular mechanisms. Nonetheless, interventional studies provide preliminary support for this causal relationship. For example, research shows that periodontal treatment significantly reduces levels of pro-inflammatory factors, such as IL-1α, IL-1β and IFN-γ, in gingival crevicular fluid (Thunell et al., 2010). Additionally, in patients with diabetes and obesity, periodontal treatment can also reduce serum concentrations of inflammatory cytokines such as TNF-α or IL-6 (Zhang et al., 2023; Guo et al., 2025). This suggests that addressing local oral inflammation can help downregulate systemic immune dysregulation, indirectly supporting the idea that persistent oral inflammation stimulates systemic immunity. However, the direct connection between these immunological improvements and changes in oral microbial composition remains unclear. Future research should employ integrated multi-omics analyses (e.g., metagenomics, transcriptomics), *in vitro* co-culture models, and mechanism-driven *in vivo* intervention studies to systematically uncover the specific ways in which oral microbiota influence immune pathways. This approach could lead to the discovery of new therapeutic targets for immune intervention in SLE.

### 4.2 Microbial translocation

Oral microbial translocation is considered another significant mechanism linking periodontitis and SLE. This process primarily occurs via intestinal and hematogenous pathways (**Figure 1**). It is worth noting, however, that this mechanism is currently based primarily on correlational studies and theoretical postulations. Direct experimental evidence is lacking, particularly functional validation from animal models.

**Figure 1:**
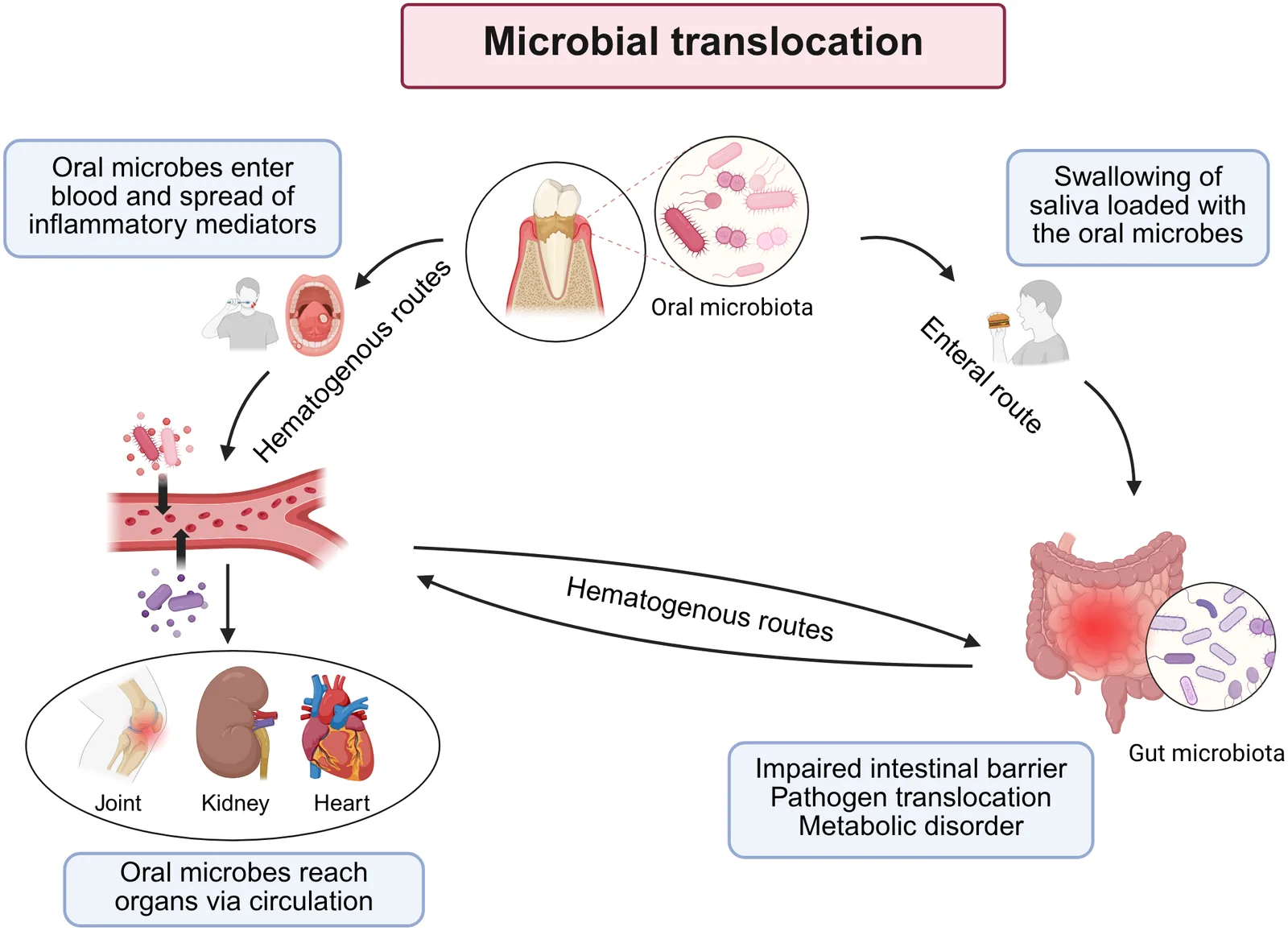
Interaction between the oral microbiota and the host involves enteral and hematogenous routes. Microbial crosstalk in (or between) the oral cavity, gut and circulatory system underpins oral microbiota-driven SLE pathogenesis. These interactions impact local and distant organs. Oral microbes enter the gastrointestinal tract via food or saliva ingestion, thereby inducing gut dysbiosis, disrupting local metabolism, and compromising the mucosal barrier. The hematogenous route represents another critical pathway for oral bacterial dissemination. Mechanical stimuli (e.g., brushing, periodontal surgeries) enable oral microbiota to breach compromised mucosal barriers, enter systemic circulation, and disseminate to distant sites (e.g., joints, kidneys, heart). When the gastrointestinal mucosal barrier is impaired, translocated oral microbes may further access systemic circulation. Created with BioRender.com.

The oral-intestinal axis serves as a vital conduit connecting the oral and gut microbiomes. Oral microorganisms, encapsulated by saliva, can traverse the gastrointestinal barrier in the form of biofilms, influencing the composition and function of the gut microbiota. In SLE patients, research indicates abnormal colonisation of multiple orally derived microorganisms in the intestines (Bao et al., 2022; Ge et al., 2024). For instance, Chen et al., 2021 discovered that untreated SLE patients’ intestines were enriched with various oral bacteria, including *Actinomyces massiliensis*, *Shuttleworthia satelles*, *Atopobium rimae*, which are often linked to oral inflammation (Downes et al., 2002; Vielkind et al., 2015; Chen et al., 2019). Similarly, Li et al., 2019 reported a significantly increased abundance of *Streptococcus* in active SLE patients, suggesting abnormal oral microbial colonisation in the gut microbiota of these individuals. Phylogenetic analyses revealed high homology between gut strains from SLE patients and those from healthy individuals, whereas gut strains from healthy controls clustered independently (Chen et al., 2021). These findings support the hypothesis that SLE patients may exhibit upper gastrointestinal barrier dysfunction, which facilitates oral microbial translocation to the gut and induces inflammatory responses. Proton pump inhibitors (PPIs) are commonly used to treat and prevent drug-related gastrointestinal damage in patients with SLE (Hailu et al., 2022; Li et al., 2022). In addition to disease-related barrier dysfunction, the use of PPIs may facilitate oral–gut microbial transmission by reducing the gastric acid barrier encountered by swallowed oral microorganisms (Jackson et al., 2016; Zhang et al., 2024b). For example, in healthy adults, Xiao et al., 2024 found that a short course of esomeprazole increased the inferred contribution of orally derived microorganisms to faecal communities. Using strain-level metagenomics in a randomized trial of healthy adults, Zhu et al., 2024 further reported that omeprazole induced stronger oral–gut strain transmission than famotidine. In patients with SLE, Li et al., 2022 found that those receiving PPIs showed higher faecal microbial diversity and a composition closer to that of healthy controls than did those not receiving PPIs. These findings suggest that acid-suppressive medication may act as a facilitator of oral-gut microbial transmission. Additionally, SLE patients exhibit widespread alterations in gut microbiota structure, notably a significantly lower *Firmicutes/Bacteroidetes* ratio compared to healthy controls (Van Der Meulen et al., 2019; Guo et al., 2020; Toumi et al., 2022). The key periodontal pathogen *P. gingivalis* further exacerbates this trend, increasing the proportion of *Bacteroidetes* while decreasing the proportion of *Firmicutes* (Nakajima et al., 2015). During the progression of periodontitis, the persistent migration of oral microorganisms into the gut and the disruption of intestinal microbial homeostasis may further increase the risk of SLE disease. However, these findings are based mainly on community association analyses using 16S rRNA sequencing, and direct evidence from microbial isolation and culture or strain-level tracking in SLE populations remains lacking. Consequently, the frequency and quantity of oral microbial translocation, along with its necessity and sufficiency in SLE pathogenesis, remain to be clarified.

On the other hand, periodontitis may also indirectly influence the progression of SLE through metabolic pathways. The salivary microbiota in individuals with periodontitis can instigate gut dysbiosis and lead to a reduction in indole metabolite production, thereby inhibiting the expression of the aryl hydrocarbon receptor (AhR) (Qian et al., 2022). As a crucial transcription factor, AhR plays a significant role in the tryptophan metabolic pathway and is closely linked to several autoimmune diseases (Wang et al., 2020; Shen et al., 2022). Tryptophan, an essential amino acid, is primarily derived from dietary sources by lactic acid bacteria and bifidobacteria and is metabolized by gut bacteria in the colon to produce indole metabolites (Roager and Licht, 2018). Indole is vital for maintaining the structure and function of epithelial cells, promoting goblet cell differentiation, and enhancing mucin production, which collectively strengthen intestinal barrier function and mitigate inflammatory responses (Li et al., 2021). AhR is involved in the activation, proliferation, and differentiation of various immune cells, including T helper cell 17 (Th17), regulatory T cells (Tregs), and B cells, making its signaling pathway a promising therapeutic target for SLE (Wu et al., 2022). These findings suggest that periodontitis may lead to systemic metabolic disorders through the salivary microbiota, with the gut microbiota serving as an intermediary mediator.

In addition to the intestinal route, the hematogenous pathway represents another significant route for the dissemination of oral microbiota (Abed et al., 2020; Gualtero et al., 2023). Studies indicate that blood flow provides a conduit for the migration of microorganisms between different sites in healthy individuals, with blood occasionally carrying DNA from microorganisms originating in various parts of the body (Tan et al., 2023). Periodontitis and invasive oral procedures offer convenient pathways for oral pathogens to enter the bloodstream (Forner et al., 2006). Oral bacteria and their associated virulence factors may enter the bloodstream through compromised gingival epithelium or oral mucosa, potentially disseminating to tissues such as the kidneys, liver, lungs, and joints, thereby triggering systemic inflammation and immune activation (Brewer et al., 2023; Liu et al., 2025). Outer membrane vesicles (OMVs) released by *P. gingivalis* are enriched in gingipains and other virulence proteins (Veith et al., 2014). In animal studies, these vesicles have been shown to deliver enzymatically active gingipains to the liver and, following local administration to periodontal tissues, to disseminate through the bloodstream to distant organs in a time-dependent manner (Seyama et al., 2020; Lv et al., 2026). Although theoretical models and some clinical observations support this pathway, further mechanistic research is required to elucidate how oral microorganisms regulate the immune response in SLE via the hematogenous route. This mechanism remains a critical research gap that requires in-depth validation.

The potential link between periodontitis and SLE via ectopic oral microbiota remains speculative, although preliminary evidence is beginning to emerge. Direct evidence of microbial translocation is difficult to obtain, as many periodontal bacteria are strict anaerobes or otherwise recalcitrant to conventional culture. Therefore, culture-based approaches should be complemented or, where appropriate, replaced by high-resolution molecular techniques, including full-length 16S rRNA amplicon sequencing, shotgun metagenomic sequencing, and, when feasible, strain-resolved genomic analyses. In parallel, detection of microbial DNA, virulence factors, extracellular vesicle components, and microbial metabolites in body fluids or affected tissues can offer corroborative evidence of translocation. Beyond correlational evidence, causal relationships need to be established through animal studies. For example, transplanting oral microbiota from periodontitis patients into germ-free lupus-prone mice could determine whether such colonization accelerates autoantibody production, proteinuria, or target-organ damage. Further, faecal microbiota transplantation could help clarify whether gut microbiota mediate the autoimmune processes triggered by oral microbes. Collectively, these lines of investigation may provide both clinical and mechanistic evidence supporting the oral dysbiosis hypothesis and clarifying its role in SLE pathogenesis.

### 4.3 Molecular mimicry

Molecular mimicry is considered a key mechanism linking infections to autoimmune diseases (Cusick et al., 2012; Qiu et al., 2019). This concept, first proposed by Damian, refers to the structural similarities between molecules from pathogens and those of the host, such as their amino acid sequences or conformations (Damian, 1964). These similarities can lead to cross-reactive immune responses, causing the immune system, which is meant to defend the body, to attack its own tissues inadvertently. Based on this, the hypothesis that “microbes trigger autoimmunity via molecular mimicry” has been advanced to elucidate the etiology of SLE (Chen et al., 2022).

Research indicates that bacterial epitopes resembling host proteins can activate T cells and B cells, driving abnormal autoantibody production (Suliman, 2024). In SLE, several autoantigens such as Ro52, Ro60, RNP-A, Sm-D3 and RNP-70K have been shown to be involved in the cross-immune response (Shmerling, 2003; Heinlen et al., 2010). Among these, the Ro60 ribonucleoprotein, one of the most common autoantigens in SLE, maintains cellular homeostasis, mediates RNA metabolism, and participates in cellular stress responses (Mahla et al., 2024). Anti-Ro60 antibodies trigger epitope spreading, generating antibodies against Ro52, Sm, and U1RNP (Deshmukh et al., 1999). Ro60 shares a 6-aa homologous sequence with the VWA domain of *P. denticola* (Obando-Pereda, 2018). Conversely, the small nuclear ribonucleoprotein 70 kDa (snRNP70), another SLE autoantigen integral to the spliceosome complex, exhibits a 7-amino acid similarity with *Bacillus cereus*, suggesting bacterial epitope mimicry may be pivotal in initiating autoimmunity (Obando-Pereda, 2018).

Antiphospholipid syndrome (APS) is an autoimmune disorder that causes thrombosis. It is characterized by vascular thrombosis and the presence of antiphospholipid antibody (aPL), such as lupus anticoagulant (LA), anticardiolipin antibody (aCL), and anti-β_2_-glycoprotein I antibody (anti-β_2_GPI) (Miyakis et al., 2006). APS was originally identified in SLE cohorts, with 20–30% of SLE patients harboring aCL (Nour-Eldine et al., 2020; Pericleous et al., 2020). 5–20% of periodontitis patients exhibit elevated serum aCL. Elevated serum levels of aCL, which target the β_2_-glycoprotein I (β_2_GPI), are linked to negative pregnancy outcomes, thrombosis, and accelerated atherosclerosis in patients with periodontitis (Schenkein and Thomas, 2020). Thus, aCL represents a key mechanistic link between periodontitis and SLE, given its elevation in both conditions. Wang et al., 2015 confirmed that *P. gingivalis*, either alone or in synergistic coexistence with *Treponema denticola* (*T. denticola*), was significantly associated with elevated serum levels of aCL and anti-β_2_GPI antibodies in patients with active SLE. Swiss-Prot database comparisons revealed homologous sequences to the β_2_GPI peptide TLRVYK within the (arginine-specific gingipain) Rgp protease of *P. gingivalis* and the phosphoglycerate kinase of *T. denticola* (Schenkein et al., 2003; Chen et al., 2009). β_2_GPI is an essential cofactor for aCL binding. Upon binding to cardiolipin, β_2_GPI undergoes a conformational change that exposes a cryptic epitope, making it the true antigenic target (Yin et al., 2018). These bacterial antigens trigger the production of anti-β_2_GPI antibodies through cross-reactivity, which promotes the generation of aCL and contributes to autoimmune attacks (Chen et al., 2009). Furthermore, aCL may target peripheral vessels in gingival tissues, inducing vasculitis or thrombosis, which can exacerbate periodontal tissue destruction due to compromised local nutrient supply. In other words, SLE patients with elevated aCL or anti-β_2_GPI antibodies may be more susceptible to periodontitis (Wang et al., 2015). The studies indicate a strong link between oral microbiota and SLE-related antibodies, though causality has not yet been confirmed in controlled animal models (**Figure 2**).

The majority of current evidence supporting the molecular mimicry hypothesis derives from bioinformatics comparisons and correlation studies in clinical research. Whether the aforementioned homologous sequences are sufficient to trigger pathogenic cross-reactive immune responses and whether such responses actually occur in humans requires further validation. Moreover, we cannot rule out potential contributions from alternative mechanisms at this time. For instance, the intense inflammatory environment induced by this bacterial infection may activate non-specific amplification through bystander effects or prolong the persistence of autoantigens. Persistence may also lead to epitope expansion, causing a broadening of immune response targets. Future studies should utilize epitope mutant infection models and compare the pathogenicity of this bacterium with that of other pathogens lacking similar epitopes to further assess the relative contribution of molecular mimicry.

**Figure 2:**
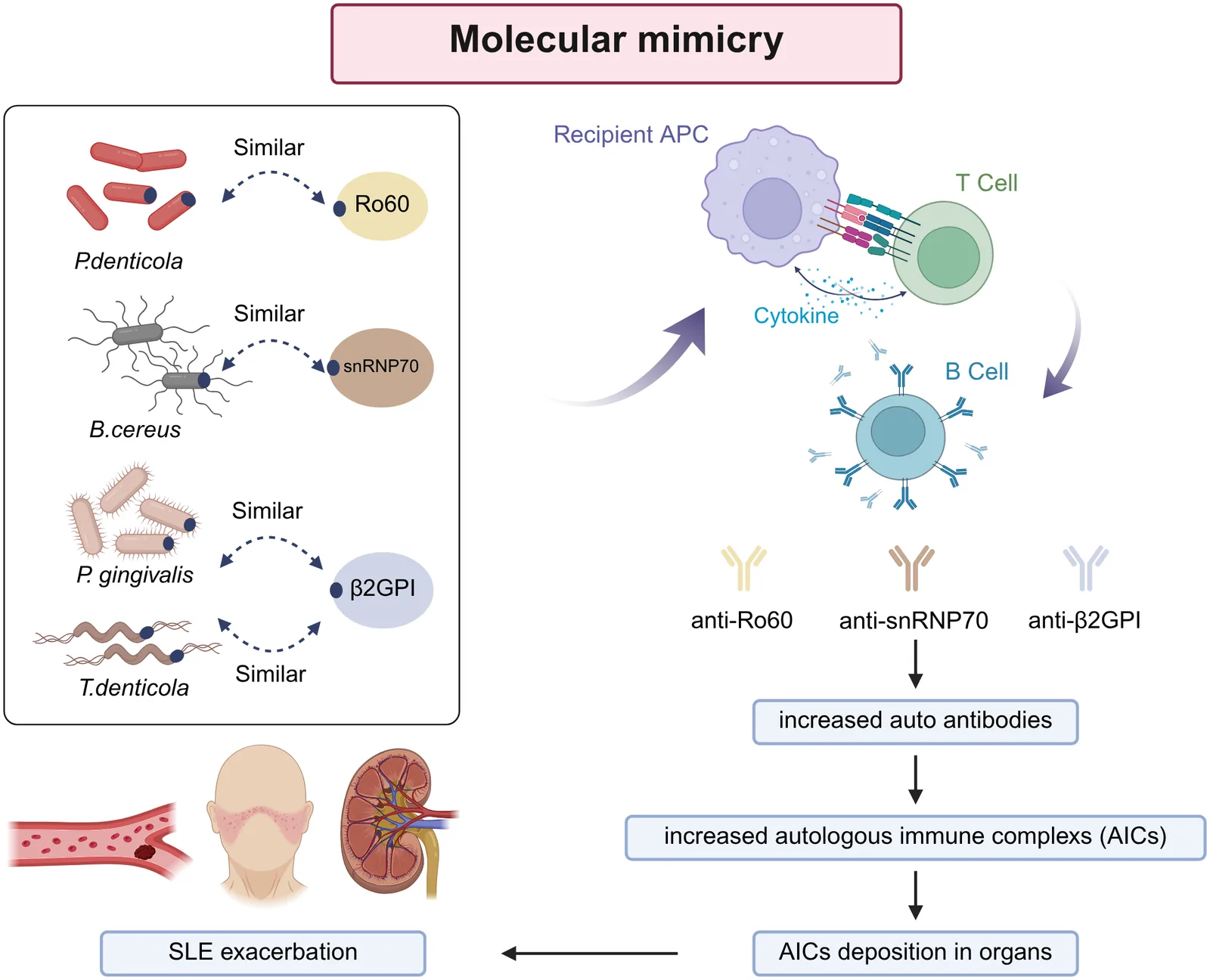
Oral microbiota trigger autoimmunity via molecular mimicry pathways. *P. denticola, B. cereus, P. gingivalis, T. denticola* trigger cross-reactive B-cell responses through molecular mimicry of human autoantigens by bacterial homologs. This process elevates autologous immune complexes, which deposit in target organs and exacerbate SLE. *P. denticola, Prevotella denticola; B. cereus, Bacillus cereus; P. gingivalis, Porphyromonas gingivalis; T. denticola, Treponema denticola*. Ro60, 60-kDa Ro ribonucleoprotein; snRNP70, small nuclear ribonucleoprotein 70 kDa; β2GPI, beta-2 glycoprotein I; APC, antigen-presenting cell. Created with BioRender.com.

## 5 Potential therapies targeting the oral microbiota for SLE

### 5.1 Maintaining good oral hygiene practices

Maintaining good oral hygiene practices, including proper brushing techniques, regular flossing, and routine dental check-ups, is crucial for overall health. Compared to healthy individuals, SLE patients often exhibit poorer oral hygiene status, and a significant positive correlation exists between disease activity and plaque index (Zhang et al., 2017). This compromised oral condition can therefore negatively impact the quality of life for SLE patients (Corrêa et al., 2018; Manzano et al., 2021). Importantly, regular periodontal treatment has been shown to effectively reduce acute-phase biomarkers in SLE patients, including C-reactive protein and erythrocyte sedimentation rate (Maybodi et al., 2022). A recent interventional study in adolescent SLE patients demonstrated that simple supragingival scaling and oral hygiene instruction not only significantly improved clinical attachment levels but also reduced disease activity (Sete et al., 2021). Therefore, maintaining good oral hygiene practices and undergoing regular dental check-ups to preserve oral microbiota balance represents a crucial strategy for preventing SLE disease progression.

### 5.2 Adjunctive use of antiseptic mouthrinses

In addition to mechanical cleaning, antiseptic mouthrinses help control plaque and modify the oral microbiota. Chlorhexidine (CHX) effectively reduces plaque accumulation (James et al., 2017). A study in healthy adults showed that seven days of CHX use altered salivary microbial composition and reduced salivary pH and oral nitrate-reducing capacity (Bescos et al., 2020). Essential-oil formulations with or without alcohol also improve plaque and gingival indices (Lynch et al., 2018). In a randomized gingivitis trial, two alcohol-containing essential-oil rinses shifted the supragingival microbiome toward that of periodontally healthy individuals after four weeks, whereas an alcohol-free formulation improved clinical indices without a comparable community shift (Min et al., 2024).

For SLE patients with periodontal disease, reducing plaque-associated microbes and local inflammation may help limit oral sources of systemic immune stimulation. The systemic effects of adjunctive mouthrinses have been investigated in RA, another systemic autoimmune disease. In a small trial, adding CHX or an essential-oil/curcumin rinse to nonsurgical periodontal therapy further reduced inflammatory and autoantibody markers (Anusha et al., 2019). Product selection and duration of use should follow dental indications, with consideration of CHX-associated staining, taste disturbance and mucosal reactions; alcohol-free formulations may be preferred in patients with xerostomia (James et al., 2017; Price et al., 2017; Sanz et al., 2020; Hong et al., 2024). The potential of mouthrinses to reduce SLE activity through oral microbiota modulation warrants further investigation in controlled clinical studies.

### 5.3 Modulating dietary patterns

Alterations in dietary macronutrients and food types have been shown to influence the oral microbiota and are associated with SLE development. Adjusting dietary patterns can help maintain oral ecological balance, such as increasing fiber and dairy intake while reducing fat and sugary food (Santonocito et al., 2022). A systematic review has indicated that a low-calorie, low-protein diet rich in resistant starch, fiber, polyunsaturated fatty acids, vitamins, minerals, and polyphenols may prevent SLE-associated cardiovascular complications (Parodi et al., 2025).

Foods such as fruits, vegetables, grains, tea, and coffee are abundant sources of plant-derived natural compounds (PDNCs), including alkaloids, lectins, flavonoids, terpenoids, and phenolic glycosides (Yang and Ling, 2025). These bioactive substances possess anti-inflammatory and immunomodulatory properties, which can reduce the production of inflammatory cytokines and intracellular signaling pathways (Balkrishna et al., 2020). By reducing autoantibody production, inhibiting pathogen proliferation, and modulating inflammatory responses, PDNCs help restore oral microbial balance and may provide therapeutic benefits for SLE. Tea, in particular, is the most widely consumed beverage in the world after water. Sayama et al., 2003 demonstrated that green tea consumption can reduce SLE risk. Epigallocatechin gallate (EGCG), the primary active polyphenol in green tea, exhibits antioxidant and anti-inflammatory effects. It inhibits NF-κB and T-cell activation and shows significant inhibitory effects on oral pathogens like *S. mutans* and *Candida albicans* (Steinmann et al., 2013; Schneider-Rayman et al., 2021). These findings indicate that PDNCs may play a key role in maintaining oral microecological balance and regulating immune function.

Vitamins are essential organic compounds that our bodies need to function normally, and most of them must be obtained through diet or other external means. Recent studies suggest that vitamin D may play a significant role in the development of autoimmune diseases by influencing the balance between the microbiota and the immune system (Murdaca et al., 2024). Vitamin D deficiency is common in SLE patients, partly due to the need to avoid sunlight. This deficiency can increase SLE susceptibility, exacerbate symptoms, impair bone health, and correlate with disease activity (Ho et al., 2024). Therefore, in addition to dietary intake, SLE patients should be appropriately supplemented with vitamin D drops. Vitamin D supplementation exerts positive effects on SLE, including inhibiting key Th17-related cytokines (e.g., IL-23 and IL-6), thereby reducing IL-17 production and increasing Treg (Predescu et al., 2025). Studies also indicate that vitamin D supplementation can alter the composition of the oral microbiota. Oral vitamin D supplementation increased the genera *Oscillospira* and *Helcococcus* while reducing *Actinobacillus*, *Ruminococcus*, *Moraxella*, *Clostridium*, *Prevotella*, *Succinivibrio*, and *Parvimonas* in bovine oral cavities (Vigors et al., 2023). Topical application of vitamin D_3_ or 1,25(OH)_2_D_3_ reduced oral pathogens such as *Enterococcus faecalis* and *Corynebacteriales* while increasing probiotics such as *Klebsiella michiganensis* (Kirkwood et al., 2024). Collectively, vitamin D may represent a potential strategy for improving oral health and SLE management by modulating oral microbial balance and promoting immune homeostasis.

Overall, the evidence above underscores the therapeutic potential of bioactive food compounds in combating oral pathogens and modulating SLE. By reshaping the oral microbiota, these compounds hold promise as novel approaches for preventing or managing SLE.

### 5.4 Supplementing probiotics

Probiotics have been shown to modulate microbial ecology through colonization of host sites, exhibiting dual capabilities: suppressing pathogen proliferation and virulence factor activity, while enhancing host immune responsiveness (Sarao and Arora, 2017). Although the role of probiotics in restoring gut microbiota balance and enhancing immune function in SLE patients has been extensively documented, their direct impact on the oral microbiota requires further investigation. Oral inoculation with *Lactobacillus plantarum* increased the levels of probiotic bacteria in the saliva, plaque, and intestinal tracts of rats, while concurrently reducing pathogenic bacteria (Wu et al., 2025). *Lactobacillus plantarum* effectively inhibits the growth of *S. mutans* and *Candida albicans*, disrupts the formation of their virulent biofilms, and influences gene expression associated with key metabolic pathways, fungal resistance, and oxidative stress (Zeng et al., 2022). Oral ingestion of *Weissella cibaria* CMS1 significantly increased salivary IgA levels and decreased TNF-α levels (Park et al., 2024). Furthermore, multiple studies have confirmed that oral *Lactobacillus* supplementation can modulate SLE progression by enhancing immunomodulatory effects (Toral et al., 2019; Manirarora et al., 2020; Yeh et al., 2021).

In summary, probiotics have the potential to benefit SLE by modulating oral microbiota balance, suppressing pathological inflammation, and enhancing immune regulation. However, their definitive therapeutic efficacy warrants further validation through robust clinical studies with rigorous designs.

## 6 Conclusions and future perspectives

The interplay between SLE and periodontitis highlights the complexity and bidirectionality of host-microbiota interactions in autoimmune diseases. Research has confirmed that SLE patients exhibit characteristic oral dysbiosis, which is marked by altered microbial diversity and an abnormal enrichment of pathogenic bacteria. This review systematically proposes that mechanisms such as cytokine-mediated immune-inflammatory amplification, microbial translocation, and molecular mimicry may be critical pathways linking the local periodontal inflammation to autoimmunity. There exists a dynamic and persistent interaction between the oral and systemic immune systems. The inherent immune dysregulation in SLE can further destabilize the oral microbiome, while oral dysbiosis may exacerbate systemic inflammation and autoimmune responses. This creates a vicious cycle of “inflammation-dysbiosis-immune activation,” which complicates disease management significantly. Therapeutically, interventions targeting the oral microbiota show considerable potential as novel adjunctive strategies for managing SLE. Approaches such as periodontal therapy and probiotic administration may help partially restore both oral and systemic immune homeostasis.

However, several critical challenges remain in this field. Firstly, much of the current evidence comes from cross-sectional studies, making it difficult to establish causality or temporal relationships between periodontitis and SLE pathogenesis. Beyond observational analyses, animal models provide an indispensable platform for establishing causal relationships and validating mechanistic hypotheses. Mao et al., 2025 recently established the first murine model of concurrent SLE and periodontitis, showing that SLE significantly aggravates periodontal inflammation and alveolar bone loss. This dual-disease model offers a valuable tool for investigating the causal mechanisms underlying SLE-associated periodontitis and for evaluating potential intervention strategies. Secondly, the effects of commonly used SLE medications, such as glucocorticoids and immunosuppressants, on the oral microbiome and periodontitis are still unclear, leading to potential biases in interpretation. More importantly, research into the specific molecular mechanisms by which periodontitis drives SLE pathogenesis is still in its infancy. Understanding how microbial metabolites and virulence factors regulate immune cell function and cause target organ damage demands further exploration at the molecular and cellular levels. Importantly, empirical evidence demonstrating microbial translocation to SLE target organs, such as the kidneys and joints, and their direct involvement in tissue damage is notably lacking.

To advance clinical translation and application in this field, future efforts should focus on several directions: First, integrating multi-omics technologies (such as metagenomics, metabolomics, transcriptomics, and single-cell sequencing) to extensively elucidate the compositional and functional alterations in SLE-associated oral microbiomes. This research could also explore the feasibility of using these alterations as non-invasive biomarkers for SLE diagnosis or assessment of disease activity. Secondly, prospective cohort studies should be conducted to clarify the predictive value of periodontitis and oral dysbiosis in forecasting SLE disease risk. Lastly, randomized controlled trials should be designed to evaluate the efficacy of periodontal treatment and oral microbiome modulation in improving SLE disease activity and prognosis. This would provide new intervention targets and an evidence-based rationale for comprehensive SLE management.
